# Superconductivity suppression and bilayer decoupling in Pr-substituted YBa_2_Cu_3_O_7−*δ*_

**DOI:** 10.1073/pnas.2536919123

**Published:** 2026-05-13

**Authors:** Jinming Yang, Zheting Jin, Siqi Wang, Camilla M. Moir, Mingyu Xu, Brandon Gunn, Rourav Basak, Joshua R. Evans, Xian Du, Zhibo Kang, Keke Feng, Makoto Hashimoto, Donghui Lu, Jessica L. McChesney, Martin Sundermann, Hlynur Gretarsson, Shize Yang, Weiwei Xie, Alex Frano, Sohrab Ismail-Beigi, M. Brian Maple, Yu He

**Affiliations:** ^a^Department of Physics, Yale University, New Haven, CT 06511; ^b^Department of Applied Physics, Yale University, New Haven, CT 06511; ^c^Department of Physics, University of California, San Diego, CA 92093; ^d^Department of Chemistry, Michigan State University, East Lansing, MI 48824; ^e^Stanford Synchrotron Radiation Lightsource, Stanford Linear Accelerator Center National Accelerator Laboratory, Menlo Park, CA 94025; ^f^Advanced Photon Source, Argonne National Laboratory, Lemont, IL 60439; ^g^PETRA III, Deutsches Elektronen-Synchrotron, Hamburg 22607, Germany; ^h^Max Planck Institute for Chemical Physics of Solids, Dresden 01187, Germany; ^i^Aberration Corrected Electron Microscopy Core, Yale University, West Haven, CT 06516

**Keywords:** superconductivity, cuprate, photoemission, rare earth element

## Abstract

The mechanism behind superconductivity suppression induced by Pr substitutions in YBa_2_Cu_3_O_7−*δ*_ (YBCO) has been a mystery since its discovery: in spite of being isovalent to Y^3+^ with a small magnetic moment, it is the only rare-earth element that has a dramatic impact on YBCO’s superconducting properties. Using angle-resolved photoemission spectroscopy (ARPES) and DFT+U calculations, we uncover how Pr substitution modifies the low-energy electronic structure of YBCO. Contrary to the prevailing Fehrenbacher–Rice (FR) and Liechtenstein–Mazin (LM) models, the low-energy electronic structure contains no signature of any *f*-electron hybridization or additional *f*-state Fermi surface sheets. Yet, strong electron doping is observed primarily on the antibonding Fermi surface. Meanwhile, we reveal major electronic structure modifications to Cu-derived states with increasing Pr substitution: a pronounced CuO_2_ bilayer decoupling and enhanced hopping along the CuO chain, implying indirect electron-release pathways beyond simple 4*f* state ionization. Our results challenge the long-standing FR/LM mechanism, and establish Pr substituted YBCO as a potential platform for exploring correlation-driven phenomena in coupled 1D–2D systems.

## Significance

For decades, it has been a puzzle why praseodymium—unlike other rare earth substitutions—so effectively suppresses superconductivity in YBa_2_Cu_3_O_7−*δ*_. Prevailing theories attributed this to a 4*f*–2p hybridization effect depleting holes on the CuO_2_ plane. Our work challenges this view: the hypothesized low-energy *f*-electron mechanism is absent up to 28% substitution with 50% $T_{c}$ suppression. Through angle-resolved photoemission spectroscopy and first-principles calculations, we reveal remarkably efficient hole reduction of 1.5$e^{-}$/Pr for surface and 0.5$e^{-}$/Pr for bulk to the superconducting plane. Meanwhile, Pr substitution substantially diminishes the CuO_2_ plane–plane interaction, which implies the existence of additional charge release channels beyond simple 4*f* electron ionization.

**Fig. 1.**
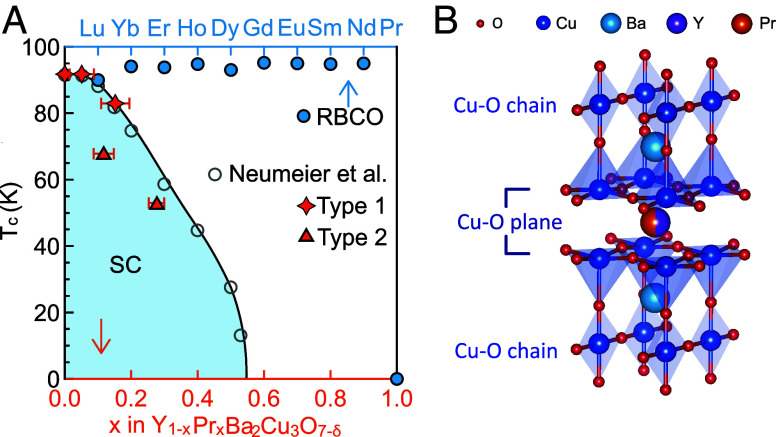
Pr substituted YBCO and REBa_2_Cu_3_O_7−*δ*_ (RBCO) superconducting transition and crystal structure of Pr substituted YBCO. (*A*) Rare earth substitution effects in YBCO. Blue circles: RBCO superconducting transition temperature (adapted from ref. 4). Gray circles (adapted from ref. 37), orange stars (Type 1), and triangles (Type 2) : $T_{c}$ as a function of Pr substitution. (*B*) Crystal structure of Pr substituted YBCO.

Elemental substitution is a powerful route to tune superconductivity in cuprate superconductors. In the pair-breaking theory of Abrikosov and Gor’kov, the depression of the superconducting transition temperature ($T_{c}$) with the concentration of RE solute in a conventional spin-singlet superconductor is predicted to scale with the de-Gennes factor of the RE ion and the square of the strength of the exchange interaction between the localized moments and conduction electron spins (1–3). Surprisingly, the substitution of most rare-earth elements for Y in YBCO has little effect on $T_{c}$ (4) (Fig. 1), suggesting that magnetic pair-breaking is very weak in these systems. Praseodymium (Pr) stands out as a striking exception: Even partial substitution of Pr for Y leads to a rapid suppression of superconductivity (5–7). Pr is unique among the rare-earth series in having both one of the smallest de Gennes factors and a putatively less localized 4*f* state, implying stronger hybridization with the conduction electrons (8–11). This stronger hybridization motivated extensions of the original pair-breaking theory to include hybridization-induced exchange interactions, but even these models fail to fully explain the unusually strong suppression of $T_{c}$ by Pr substitution (11–14). Understanding why Pr substitution is uniquely destructive to superconductivity thus remains a key open question, and resolving this puzzle may provide insights into the pairing mechanism in cuprates.

In view of the inability of magnetic pair-breaking to fully account for the strong depression of $T_{c}$ of Pr substituted YBCO, the depression of $T_{c}$ has been attributed in large part to the depletion of hole carriers. Thus, Pr substituted YBCO appears to be an underdoped system similar to oxygen-deficient YBCO, a viewpoint that is supported by evidence for the formation of a pseudogap in transport measurements (11, 14–19) and Ca^2+^ countersubstitution experiments (20, 21). However, electron doping appears unlikely due to the nominal isovalence of Pr^3+^ and Y^3+^. This led to proposals of hole localization (22–30). Such localization of holes could occur when Pr 4$f_{z(x^{2}-y^{2})}$ orbitals are hybridized with O 2p_*π*_ states to form new hole bands at the Fermi level ($E_{F}$), as suggested by FR (31) and LM (32) models. The models were based on the hypothesized distinguishing feature of Pr among rare earth elements: Its 4$f_{z(x^{2}-y^{2})}$ states lie at the $E_{F}$; however, direct experimental evidence for such low-energy 4*f* contributions remains lacking.

Recently, Pr substituted YBCO was found to host long-range three-dimensional charge order (CO) (33) with in-plane CO at the Mott limit (34), where FR and LM model pictures are proposed to be relevant. Moreover, rare-earth infinite-layer nickelates *RE*NiO_2_—sharing structural similarities with Pr substituted YBCO—exhibit parallel debates about *f*-state involvement (35), but first-principles calculations indicate the absence of low-energy *f* states (36). These developments underscore the critical need to resolve how Pr substitution modifies the electronic structure in these archetypal superconducting transition metal oxides. Here, we employ angle-resolved photoemission spectroscopy (ARPES) to directly probe the low-energy electronic structure, which is complemented by density functional theory (DFT)+U calculations and nonresonant inelastic X-ray scattering. Together, these techniques allow us to address the following key questions: i) Are there f-states on the Fermi surface; ii) how Pr substitution alters the low-energy electronic structure; and iii) if $T_{c}$ is indeed dictated by hole localization in Pr substituted YBCO.

## Results

We investigate pristine YBCO single crystals ($T_{c}=91$ K) alongside four Pr substituted variants with $T_{c}$’s of 91 K, 84 K, 63 K, and 53 K, where Pr content is found to be 5%, 15%, 12%, and 28% by energy-dispersive X-ray spectroscopy (EDX) measurements, respectively. As shown in Fig. 1*A*, the measured samples are categorized into two types. Type 1 follows the typical Pr substituted YBCO phase diagram, while type 2 shows lower $T_{c}$ at the same Pr content. The existence of type 2 implies potential additional $T_{c}$ suppression mechanisms in these samples. To elucidate the superconductivity suppression mechanism, we conduct ARPES measurements of the electronic structure.

**Fig. 2.**
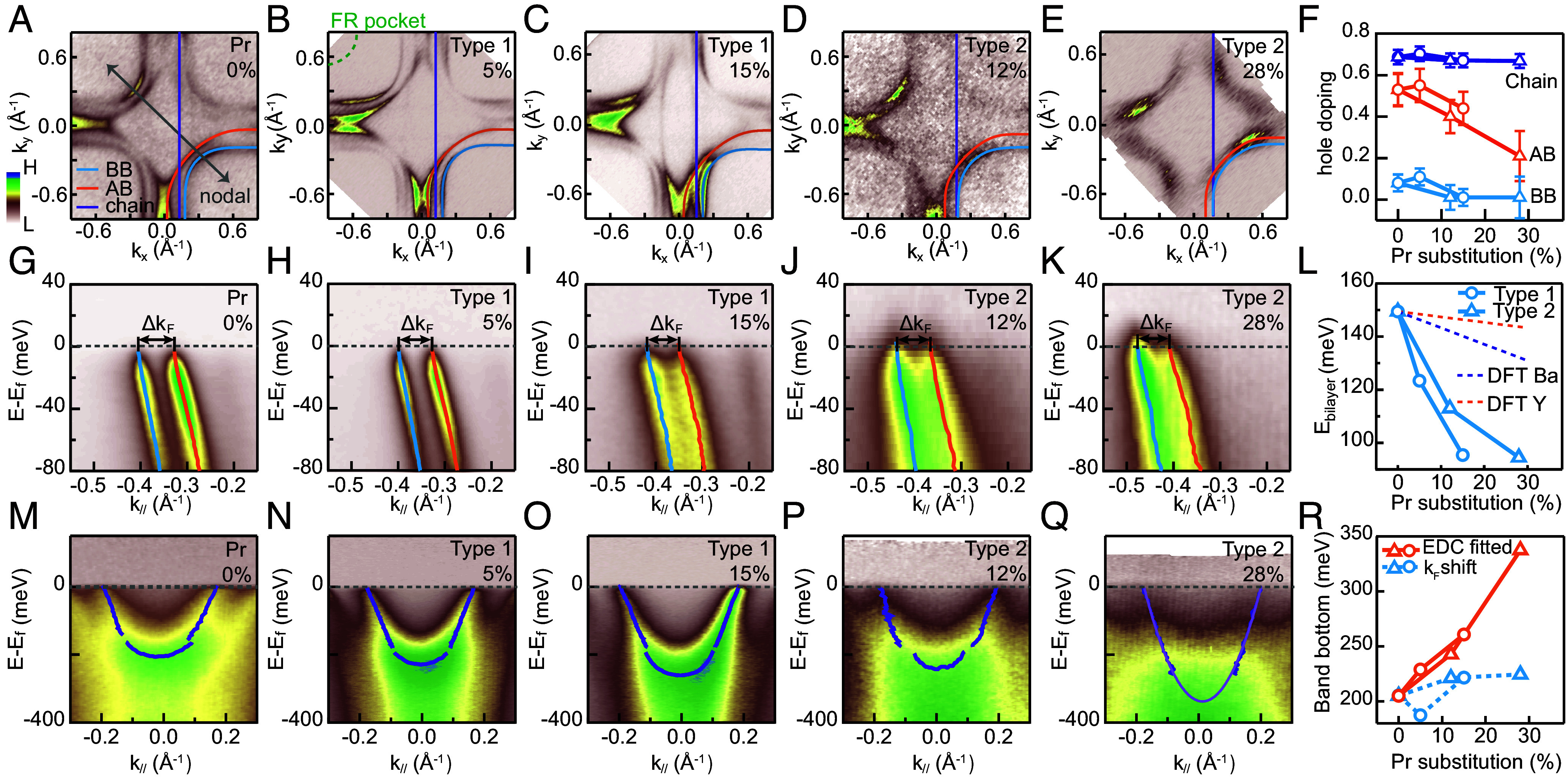
ARPES measured electronic structure of Pr substituted YBCO. (*A*–*E*) Fermi surfaces with extracted Fermi momenta. (*F*) Hole doping level evolution for AB, BB and chain with Pr substitution. Circles (triangles) represent type 1 (type 2) samples. (*G*–*K*) Nodal cuts at 51 eV highlighting plane bands with fitted dispersions. (*L*) Bilayer splitting energy evolution with Pr substitution. (*M*–*Q*) Nodal cuts at 43 eV highlighting chain band with fitted dispersions. (*R*) Band bottom position evolution with Pr substitution. Circles (triangles) represent type 1 (type 2) samples. Orange curves are extracted directly from EDC fitting. Blue dashed lines are band bottom position evolution expected from pure charge doping effects.

Pristine YBCO exhibits three bands crossing $E_{F}$: the bonding (BB) and antibonding (AB) bands from the CuO_2_ bilayer, and a quasi-1D band from the CuO chain (38–54). By exploiting the photoemission dipole transition matrix element effects (see *SI Appendix*, Fig. S10), we selectively enhance either plane-derived or chain-derived bands for detailed analysis. In FR/LM models, Pr 4f-O $2p_{\sigma }$ hybridization should generate an additional dispersive band with a hole pocket centered at (π,π) (dashed line, Fig. 2*B*) (32). However, ARPES measurements (Fig. 2*B*–*E*) show no such Pr-derived pocket at the Brillouin zone corner for all samples with up to 28% Pr substitution. This absence is further confirmed by nodal cuts, which reveal no low-energy 4f-related bands. Neither is there any visible indication of *f*-hybridization-induced anti-crossing near the Fermi level $E_{F}$ (*SI Appendix*, Fig. S11).

Hole reduction in the CuO_2_ plane within the FR/LM picture occurs via hole transfer to the local Pr–O bonds. The absence of low-energy 4f bands implies that any hole doping reduction must directly occur on CuO_2_ planes or CuO chains. Hole concentration changes are quantified by fitting a global tight-binding model to both BB and AB bands in the 0 to 50 meV binding energy range (55) (also see *SI Appendix*, Fig. S9 and Text S1). The model is defined as

$$\begin{array}{cc} E_{\pm } & =\epsilon_{\pm }-2t_{\pm }\left(\cos k_{x}+\cos k_{y}\right)\\ & -4t_{\pm }^{\prime }\cos k_{x}\cos k_{y}-2t_{\pm }^{\prime \prime }\left(\cos2k_{x}+\cos2k_{y}\right), \end{array}$$

where ± denotes the bonding (BB) (−) or antibonding (AB) (+) band (see *SI Appendix*, Text S1 for details). Hole doping levels for the CuO_2_ planes and CuO chains are extracted by applying Luttinger’s theorem to their respective Fermi surface sheets. Previous ARPES studies of pristine YBCO were confounded by surface hole-doping saturation effects (39, 40, 42–44, 46, 48–51, 53, 54), with electron doping only observed through surface alkali metal dosing (49, 51). Remarkably, in as-cleaved Pr substituted YBCO, we observe major electron doping effects manifested through a systematic shrinkage of hole pockets from the AB/BB bands (Fig. 2*A*–*E*). Fig. 2*F* reveals that electron doping occurs primarily on the AB band and moderately on the BB band of the CuO_2_ bilayer, while the chain band shows minimal change. Notably, type 2 samples experience greater electron doping than type 1 samples, despite lower global Pr concentration, which unexpectedly leads to exacerbated $T_{c}$ suppression.

The disproportionate hole-doping of the AB/BB bands also implies an appreciable change to the CuO_2_ bilayer energy splitting with Pr substitution (Fig. 2*A*–*E*). Along the nodal direction (Fig. 2*G*–*K*), bilayer splitting energy measured with $\hbar v_{F}\Delta k_{F}$ is substantially suppressed by over 30% from pristine to both types of Pr substituted YBCO (Fig. 2*L*), signaling rapid electronic decoupling of the CuO_2_ bilayer. While first-principles calculations (dashed lines, Fig. 2*L*) show moderate decoupling depending on different Pr substitution sites, the experimental reduction exceeds theoretical predictions even more, indicating possible additional decoupling mechanisms.

Unlike the planar bands, the CuO-chain band shows only weak electron doping (purple lines, Fig. 2*F*). However, dramatic changes in the effective mass are observed. As shown in Fig. 2*M*–*Q*, chain bands steepen progressively with Pr substitution. This is characterized by a systematic shift of the chain band bottom toward higher binding energy. As shown in Fig. 2*R*, this energy shift (orange) far exceeds what the small electron doping can induce. Notably, quasiparticle coherence—especially on the chain band—is suppressed in type 2 samples (Fig. 2*M*–*Q*), suggesting disproportionately high Pr-induced disorder effects on the chain band, which will be discussed later.

**Fig. 3.**
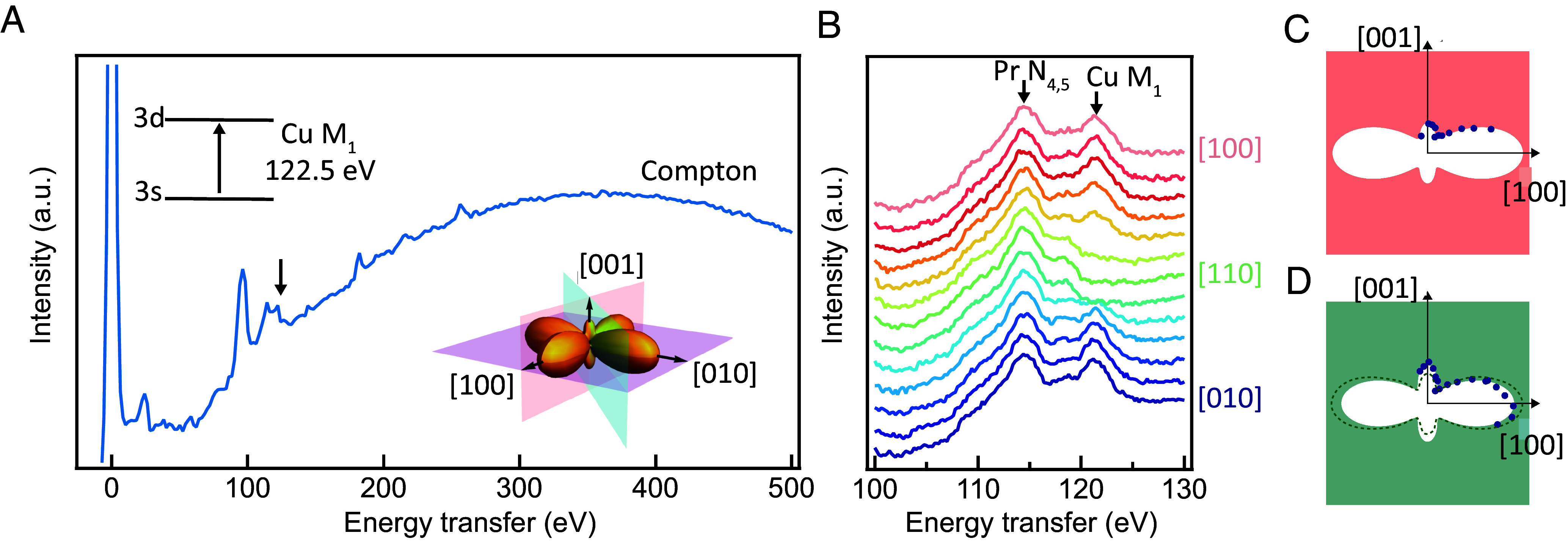
Cu 3d orbital imaging with nonresonant inelastic X-ray scattering. (*A*) Energy loss spectrum measured with an incident energy of 9,690 eV on nominally 80% Pr substituted YBa_2_Cu_3_O_7−*δ*_ (nonsuperconducting) at 20 K along the [100] direction. (*B*) In-plane angular dependence of the Cu M_1_ transition intensity as a direct measure of the in-plane angular profile of the Cu 3$d_{x^{2}-y^{2}}$ orbital. Orbital lobe profile along the xz plane for (*C*) 80% Pr substituted YBa_2_Cu_3_O_7−*δ*_ and (*D*) fully oxygenated pristine YBa_2_Cu_3_O_7−*δ*_ ($T_{c}=90$ K). The black dotted line denotes the orbital shape of the Pr-doped sample for ease of comparison.

To understand how the $e_{g}$ orbital of the planar copper evolves with Pr-substitution of Ba, we perform angle-dependent nonresonant inelastic X-ray scattering to trace the relative anisotropy of the in-plane (3$d_{x^{2}-y^{2}}$) and the out-of-plane (3$d_{z^{2}}$) orbitals. This technique is based on the principle that the angle-dependent transition probability for the dipole-forbidden 3s to 3d excitation (M_1_ edge) exactly follows the angular profile of the 3d orbital (56). As shown in Fig. 3*A*, the Cu M_1_ and Pr N_4,5_ transitions are clearly resolved around 120 eV energy loss. Notably, the in-plane angular dependence of the Cu M_1_ transition intensity follows the lobe-structure of the 3$d_{x^{2}-y^{2}}$ hole state (Fig. 3*B*). By comparing the out-of-plane angular dependence of the M_1_ transition probability in 80% Pr substituted (Fig. 3*C*) and pristine YBa_2_Cu_3_O_7−*δ*_ (Fig. 3*D*), a clear “flattening” of overall Cu $e_{g}$ orbital occurs with heavy Pr-doping. This indicates lower energy and fewer hole states for the 3$d_{z^{2}}$ orbital with Pr substitution, henceforth offering a direct wavefunction view of the associated bilayer decoupling, consistent with the bilayer splitting energy reduction seen in ARPES.

## Discussion

**Fig. 4.**
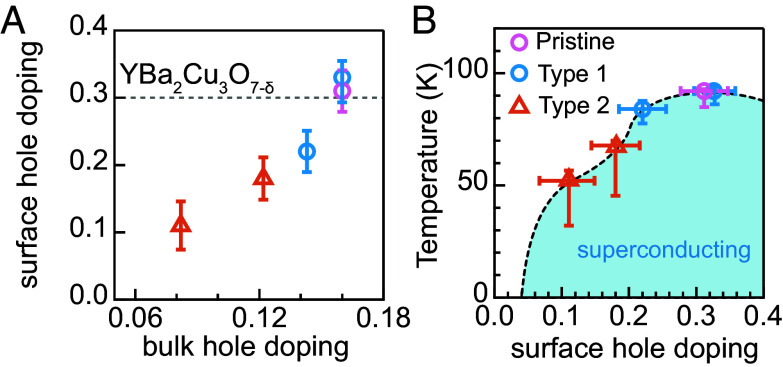
Electron doping and superconductivity suppression in Pr substituted YBCO. (*A*) Surface hole doping level dependence on the bulk doping level for Pr substituted YBCO (markers) and YBCO with hole doping controlled by oxygen content (gray dashed line from ref. 46). The bulk hole doping level for Pr substituted YBCO is obtained by comparing $T_{c}$ with the oxygen content controlled YBCO phase diagram in ref. 57. (*B*) Superconducting transition temperature against surface hole doping level derived in this work.

Our observations suggest that electron doping to the CuO_2_ plane is the major mechanism behind superconductivity suppression in the system. First, we note that the surface doping extracted by ARPES here is not directly comparable to bulk doping due to the lack of neutral cleavage planes in YBCO (39, 41, 43, 44, 53, 54).^*^ Such a polar surface will undergo charge redistribution to avoid the polar catastrophe (58). As a result, the surface hole doping level of cleaved YBCO was found to remain approximately constant at 0.3 regardless of its oxygenation level (59). The additional hole doping compared to the bulk is believed to be associated with the chain band charging state (46, 48, 50, 54, 60). In remarkable contrast, on the as-cleaved surface for Pr substituted YBCO, the surface hole doping level changes correspondingly with the bulk doping level (Fig. 4*A*). Fig. 4*B* plots $T_{c}$ against the ARPES-derived surface hole doping, which qualitatively tracks the canonical bulk phase diagram. We emphasize that this replication of the dome shape from ARPES-derived hole-doping levels strongly suggests that, regardless of the sample types, electron doping dominates the $T_{c}$ suppression. Intriguingly, the long-range c-axis charge order then occurs at a Pr substitution (30%) qualitatively close to the equivalent charge order hole doping in oxygenated YBCO.

The absence of FR states at $E_{F}$ compels a reassessment of Pr’s effect on the valence states of other elements. One key structural effect with Pr-substitution is the flattening of the CuO_5_ pyramid, which is generally considered to reduce the Cu valence by lowering the Cu 3$d_{x^{2}-y^{2}}$ site energy (61, 62). Another possible origin of additional electron doping is from partial Pr^3+^ to Ba^2+^ substitution, which was suggested in earlier studies but without conclusive experimental evidence (63–67). The presence of type 2 samples may relate to partial Ba-site substitution, which is evidenced by composition analysis, single crystal X-ray diffraction refinement (*SI Appendix*, Table S2 and Fig. S13), scanning tunneling microscopy (*SI Appendix*, Fig. S14), and disorder-induced chain band broadening effects (further discussed in DFT calculations). To understand the potential site-dependent Pr substitution effect, we model three systems: pristine YBa_2_Cu_3_O_7_, Y_0.67_Pr_0.33_Ba_2_Cu_3_O_7_ (Y-site substitution), and YBa_1.67_Pr_0.33_Cu_3_O_7_ (Ba-site substitution), using 3×2×1 supercells with two Y/Ba atoms replaced by Pr. Structure relaxations reveal the lowest-energy configuration features Pr atoms aligned along Cu-O chains with antiferromagnetic (AFM) ordering. Ferromagnetic (FM) configurations cost ∼2 meV/Pr, which is consistent with the observed ∼17 K AFM transition in PrBa_2_Cu_3_O_7_ (10). (Computational details in *Methods* and *SI Appendix*, Text S2).

**Fig. 5.**
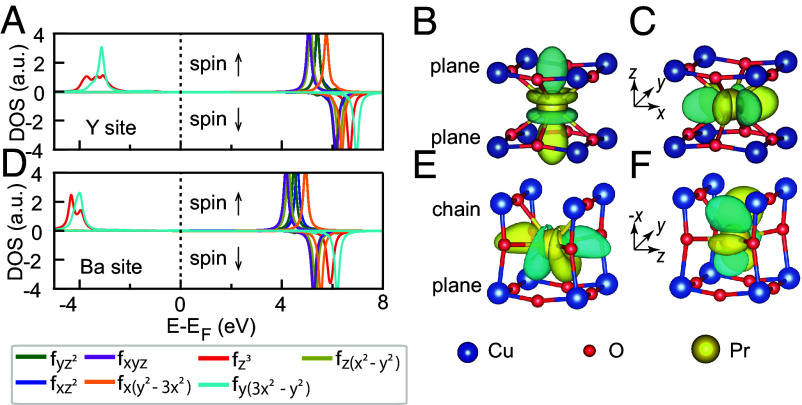
Pr *f*-orbitals predicted by DFT calculations. (*A*) The ground-state projected density of states (DOS) of Pr *f*-orbitals on the Y site. Fermi energy is set to be the reference energy on the horizontal axis. (*B* and *C*) Corresponding Wannier function isosurfaces of the occupied orbitals (*B*) $f_{z^{3}}$ and (*C*) $f_{y(3x^{2}-y^{2})}$, where blue and yellow represent positive and negative values, respectively. The isosurface level is chosen at 20% of the maximum absolute value. The xyz coordinates represent the local coordinates used to define the orbitals of Pr. (*D*–*F*) DOS and Wannier functions of Pr *f*-orbitals on the Ba site. The local coordinates are rotated compared to (*A*–*C*).

After full structural relaxation of both Y-site and Ba-site substituted systems, we find a striking result: the ground state of neither configuration exhibits occupied $4f_{z(x^{2}-y^{2})}$ orbitals (Fig. 5)—the essential component for FR singlet formation. Instead, DFT+U reveals that Pr 4f electrons predominantly occupy the $4f_{y(3x^{2}-y^{2})}$ and $4f_{z^{3}}$ states, positioned approximately 4 eV below $E_{F}$. This orbital configuration aligns perfectly with crystal field expectations: as shown in Fig. 5 *C* and *E*, these specific *f*-orbitals minimize energy by orienting electron density away from neighboring oxygen atoms. In addition, Pr clearly adopts a 3+ valence at both Y and Ba sites ($4f^{2}$ local configuration featuring a large energy gap).

To reconcile our findings with the LM model, we enforced occupation of the $4f_{z(x^{2}-y^{2})}$ and $4f_{z^{3}}$ orbitals in Y_0.67_Pr_0.33_Ba_2_Cu_3_O_7_ using occupation matrix control (68). This constrained calculation converges to a metastable state ∼140 meV/Pr above the ground state energy, which indeed displays FR bands (31) through antibonding Pr $4f_{z(x^{2}-y^{2})}$-O 2p hybridization (*SI Appendix*, Text S2). Extending this analysis to full Y-site substitution, we reproduced the LM-predicted band structure for PrBa_2_Cu_3_O_7_ (32) (*SI Appendix*, Text S2). Crucially, this configuration remains metastable, lying ∼328 meV/Pr above the true ground state. As with lower substitution concentrations, the authentic ground state features occupied $4f_{y(3x^{2}-y^{2})}$ and $4f_{z^{3}}$ orbitals, rather than the low-energy *f*-states required for FR/LM hybridization. These results challenge the FR/LM mechanism as an explanation for superconductivity suppression in the low-doping regime.

**Fig. 6.**
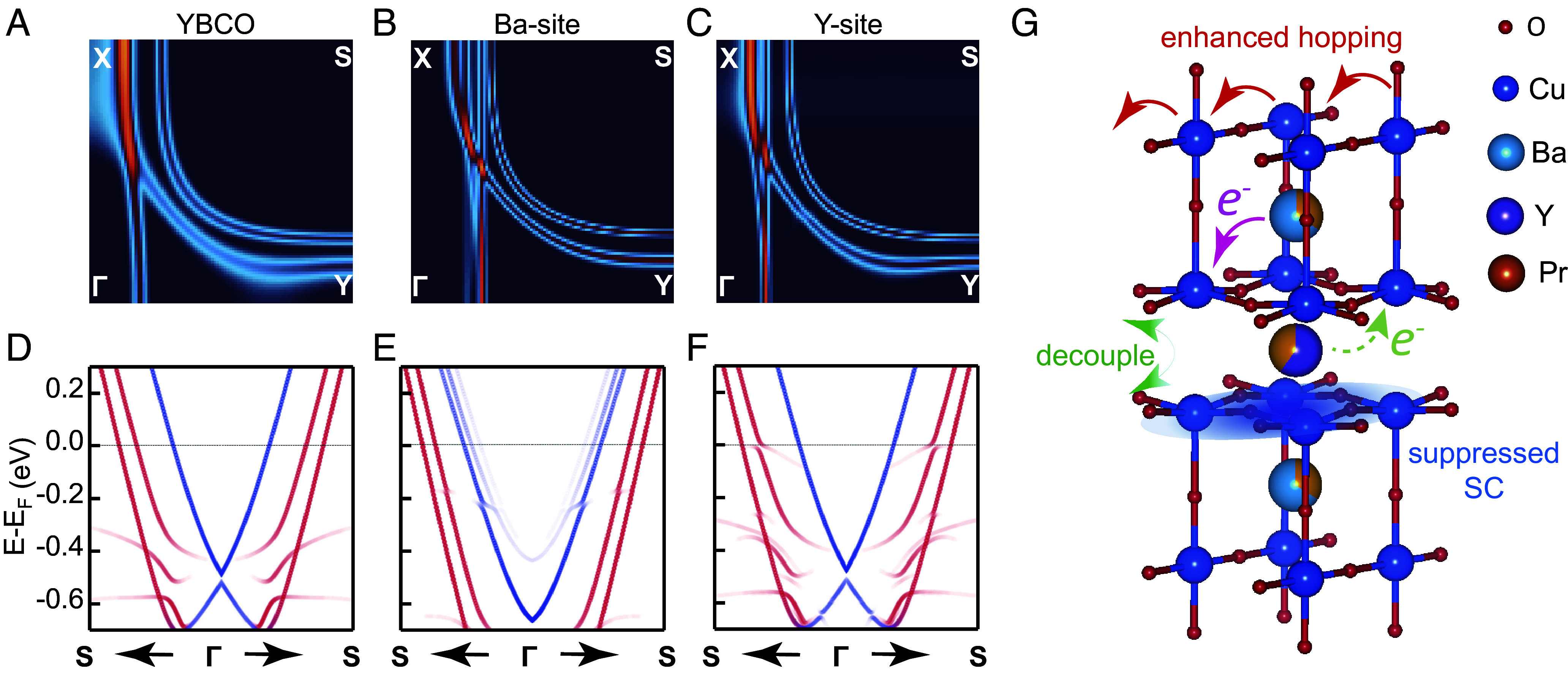
Dual site occupancy effects of Pr substitution. (*A*–*C*) DFT Fermi surfaces for pristine, Ba-site 33% and Y-site 33% Pr substitution, respectively. (*D*–*F*) Nodal band structures. Blue (red) lines represent projections on chain Cu $d_{z^{2}}$ (planar Cu $d_{x^{2}-y^{2}}$) orbitals. (*G*) Schematic drawing of Pr substitution effects on two preferential sites.

Experimentally, Pr substitution nonetheless alters the low-energy electronic structure. Most notably, it leads to a strong decoupling of the CuO_2_ bilayers and enhances hopping along the chains, as shown in Fig. 2. In Fig. 6, we present the DFT-calculated electronic structures of pure YBCO and Pr substituted YBCO at Ba and Y sites. Comparing YBCO (Fig. 6 *A* and *D*), Ba-site Pr substituted YBCO (Fig. 6 *B* and *E*), and Y-site Pr substituted YBCO (Fig. 6 *C* and *F*), Pr substitution introduces three major changes in the electronic structure: i) A reduction of bilayer splitting (more significant for Ba-site substitution, and to a lesser extent for Y-site substitution); ii) The emergence of a cascade of shadow chain bands in the Ba-site substituted sample; iii) Heavy electron doping, directly observed from the increased Fermi momentum of both chain and plane bands in the Ba-site substituted sample. We now discuss these changes and compare them with experimental results.

CuO_2_ bilayer interaction has been proposed as a potential factor behind the enhanced $T_{c}$ in the multilayer members within a given cuprate family. The bilayer Josephson tunneling model has been proposed as a primary superconducting pairing mechanism in such systems (69, 70), but the associated anomalous kinetic energy gain in the superconducting transition remains elusive (71). Later, it was also suggested that $T_{c}$ could be enhanced by coupling layers of strong pairing strength with layers of high phase stiffness respectively, demonstrated through both phenomenological theory and numerical studies in bilayer and multilayer systems (72–74). In both scenarios, interlayer coupling helps enhance $T_{c}$. Given the relatively large bilayer coupling in YBCO, it serves as an excellent model system to investigate how interlayer coupling may be correlated with superconductivity. However, very few studies have demonstrated the ability to tune this coupling (51). Here, both our experimental results and DFT calculations show a reduction in bilayer splitting induced by Pr-substitution (Fig. 2*L*). The slower trend predicted from DFT indicates an additional correlation effect is likely at play, consistent with earlier reports in oxygenated YBCO (51). Under this scenario, the disproportionate charge doping effect on the bonding vs. antibonding bands (Fig. 2*F*) also predicts a robust superconducting pairing gap (due to a nearly half-filled bonding band) with a weakening superfluid density (due to hole depletion on the antibonding band), which can be tested by further magnetic penetration depth measurements.

Now we turn to the loss of quasiparticle coherence in the chain band. First-principles supercell calculations show that local structural distortions induced by Ba-site Pr substitution substantially reduce the lateral spacing between CuO chains by about 0.3 Å, which in turn causes a ∼125 meV shift in the onsite energy of the chain Cu $d_{z}^{2}$ Wannier orbital. This results in the appearance of shadow chain bands, as shown in Fig. 6 *B* and *E*, which appear as smeared spectra experimentally (Fig. 2 *P* and *Q*). In contrast, Y-site Pr substitutions cause an order of magnitude smaller changes to the structure and onsite energy, leaving the chain bands largely unaffected, as seen in Fig. 6 *C* and *F*. Meanwhile, the CuO_2_ planes are less sensitive to Pr substitution due to their structural rigidity, as two additional oxygen atoms around each Cu atom reinforce the lattice, limiting its ability to distort. Pr substitutions cause atom displacements of up to 0.05 Å in the CuO_2_ planes, leading to onsite energy changes of up to ∼25 meV, again, an order of magnitude smaller than in the chains. These findings show that 1D CuO chains are far more susceptible to structural disruption than their 2D counterparts.

To conclude, we present a comprehensive study of how Pr substitution modifies the electronic structure of YBCO. Fig. 6*G* summarizes the main findings of this work. First, superconductivity suppression in Pr substituted YBCO arises from substantial electron doping to the antibonding band. Our combined experimental and first-principles results also reveal the limitation of the FR/LM mechanism of hole localization mechanism, as no additional FR bands are seen near the Fermi level. Our calculations show that this model is a high-energy metastable configuration, whereas the predicted ground state for Pr replacement at the Y-site is firmly Pr^3+^ with no predicted additional electron donation to the antibonding band. We propose several possible explanations to explain the strong electron donation and $T_{c}$ suppression in Pr substituted YBCO: a) if the only effect of Pr substitution is to replace either Y or Ba and the DFT+U predictions for the ground-state electronic structure are correct, then the extent of electron donation must be linked with the fraction of Ba site replacement, which can be verified with high-resolution XRD; b) incorporation of Pr has other structural effects (e.g., disorder, vacancies, interstitial ions) that act as electron donors; c) there are novel many-body effects, missing from DFT+U theory, due to Pr replacement on the Y site that lead to a reduction of the effective hole count in the antibonding band (e.g., a many-body renormalization of spectral weight). In addition to the above, we observe a rapid decoupling of the CuO_2_ bilayers and enhanced electron hopping along the CuO chain, indicating significant modifications of both plane–plane and chain–plane coupling. The chain band shows extreme sensitivity to potential Ba-site disorder, while the planar bands remain robust. These results highlight Pr substituted YBCO as an ideal platform for investigating high-$T_{c}$ superconductivity and other correlated phenomena through site-specific electronic structure engineering of the CuO chains and CuO_2_ planes.^†^

## Materials and Methods

### Sample Preparation.

Single crystals of nominal composition Pr_*x*_Y_1−*x*_Ba_2_Cu_3_O_7−*δ*_ (11) were synthesized following the procedure outlined in ref. 75. High-purity (99.99%) Y_2_O_3_, Pr_6_O_11_, BaCO_3_, and CuO powders were used as starting materials. Postgrowth, the crystals were annealed in flowing oxygen to ensure full oxygenation and to optimize their superconducting properties. Atomic concentration was measured with Oxford instrument EDS under JEOL 6610LV scanning electron microscopy and BRUKER XFlash 5060FQ Annular EDS detector under Hitachi SU8230 UHR CFE scanning electron microscopy. The superconducting transition temperatures ($T_{c}$) were characterized via magnetization measurements using a vibrating sample magnetometer integrated in a Quantum Design DynaCool Physical Property Measurement System.

### Single Crystal X-Ray Diffraction.

Single crystal X-ray diffraction results were obtained using a Rigaku XtaLAB Mini II system and a Rigaku XtalLAB Synergy, Dualflex, Hypix single crystal X-ray diffractometer at room temperature. Crystallographic data acquisition was conducted employing ω scan methodology, utilizing Mo $K_{\alpha }$ radiation (λ=0.71073 Å) emitted from a microfocus sealed X-ray tube under operating conditions of 50 kV and 1 mA. The determination of the experimental parameters, including the total number of runs and images, was derived algorithmically from the strategy computations facilitated by the CrysAlisPro software, version 1.171.42.101a (Rigaku OD, 2023). Subsequent data reduction processes incorporated corrections for Lorentz and polarization effects. Integration of the collected data was conducted using the sphere model. An advanced numerical absorption correction was implemented, leveraging Gaussian integration across a model of a multifaceted crystal (76). Moreover, an empirical absorption correction employing spherical harmonics was applied within the SCALE3 ABSPACK scaling algorithm to refine the data further (77).

### Scanning Transmission Electron Microscopy.

Scanning transmission electron microscopy imaging and EDS analysis were carried out with a Spectra Ultra microscope operated at 300 kV with a cold field emission gun. The EDS detector was Ultra-X EDS (silicon drift detectors with a collection solid angle of 4.45 srad). The probe semiconvergence angle was set at 30 mrad with a camera length of 110 mm and a probe current of 50 Pa.

### Nonresonance Inelastic X-Ray Scattering.

The NIXS measurements were performed under ultrahigh vacuum at the High-Resolution Dynamics beamline P01 of PETRA-III at Deutsches Elektronen-Synchrotron (DESY, Germany). The incident X-ray beam energy was tuned using a Si(111) double-reflection crystal monochromator. The scattered photons were analyzed by a 3×4 array of spherically bent Si(660) crystal analyzers fixed to an energy of 9,690 eV. The energy loss spectra were measured by continuously sweeping the monochromator from 9,690 eV (the elastic line) to higher energies, thus scanning the energy transferred in the inelastic scattering process. The experimental resolution, which is estimated by the full width at half maximum of the elastic line, was measured to be ∼1.4 eV. Fixing the scattering angle to $2\theta =155^{{}^{\circ}}$ yields a momentum transfer vector $q=k_{\mathrm{in}}-k_{\mathrm{out}}\approx 9.6\;{Å}^{-1}$. All samples were polished to reduce surface defects. All NIXS spectra presented in this work were measured at 20 K and were normalized by the spectral weight of the Compton background.

### ARPES Measurement.

Synchrotron ARPES measurements were performed at beamline 5 of the Stanford Synchrotron Radiation Lightsource. A hemispherical electron analyzer (DA30, Scienta) was used. The k_*z*_ dependence was measured with photon energy varying from 30 to 80 eV (*SI Appendix*, Fig. S10). All measurements were done using linear horizontal polarization. The Fermi surface map was measured at 87 eV. The detector nonlinearity was calibrated and corrected. The chemical potential of the sample and the energy resolution of the system were determined by fitting the Fermi edge of polycrystalline gold. An energy-independent background was determined using intensity far above the chemical potential and subtracted from the data.

### First-Principles Calculations.

All DFT calculations were based on the Vienna ab initio simulation package (VASP) with the projector-augmented wave method (78). A relatively high plane-wave cutoff energy of 500 eV is used, and a relatively dense 4×6×4 k-grid is used for 3×2×1 supercells. All results come with full structural relaxation where energies and forces are converged to $10^{-6}$ eV and $10^{-3}$ eV/Å, respectively. The generalized gradient approximation (GGA) with the semilocal Perdew–Burke–Ernzerhof (PBE) functional (79, 80) is used in all calculations. In addition, we add $U_{\text{Cu}}=4$ eV for the Cu d manifold following previous theoretical works (81, 82). Varying $U_{\text{Cu}}$ between 0 and 9 eV shows little effect on the YBCO band structure for the paramagnetic state (59). For the *f*-orbitals of Pr, prior theoretical works used $U_{\text{Pr}}=5$ to 10 eV (32, 33, 83, 84). We find that varying $U_{\text{Pr}}$ within this range always shows insulating Pr bands at least 1 eV away from the Fermi level, which does not qualitatively affect our results. The results in the main text come with $U_{\text{Pr}}=8$ eV. Maximally localized Wannier functions (85) consisting of all Cu-d, O-p, and Pr-f orbitals were extracted from our DFT calculations using Wannier90 (86). To enable direct comparison with experimental ARPES measurements of the Fermi surface, we employ the standard band unfolding technique (87, 88) for all electronic structures, which projects the band structure of a large supercell onto the Brillouin zone of the primitive unit cell. This approach has been shown to qualitatively reproduce spectral intensities observed in ARPES experiments across a wide range of materials (81, 89–92).

## Supplementary Material

Appendix 01 (PDF)

## Data Availability

Source data have been deposited in FigShare (https://doi.org/10.6084/m9.figshare.30899429) (93).

## Footnotes

^*^Charge-neutral cleavage planes have recently been observed, but only in small domain size on the order of several micrometers (54). In this work, we focus on the typical cleavage plane domain that highlights the plane bands illustrated in ref. 53.

^†^All data for this work is available https://doi.org/10.6084/m9.figshare.30899429.
