# Supplementary material for: Superconductivity suppression and bilayer decoupling in Pr-substituted YBa2Cu3O7−δ
Source: Proc Natl Acad Sci U S A. 2026 May 13;123(20):e2536919123. doi: 10.1073/pnas.2536919123 (PMC13187780; doi:10.1073/pnas.2536919123)
Supplement: Supplementary file 1 — Appendix 01 (PDF) [file pnas.2536919123.sapp.pdf]

Supporting Information for

Superconductivity suppression and bilayer decoupling in Pr substituted $YBa_2Cu_3O_{7-\delta}$

Jinming Yang, Zheting Jin, Siqi Wang, Camilla Moir, Mingyu Xu, Brandon Gunn, Rourav Basak, Joshua R. Evans, Xian Du, Zhibo Kang, Keke Feng, Makoto Hashimoto, Donghui Lu, Jessica McChesney, Martin Sundermann, Hlynur Gretarsson, Shize Yang, Wei-Wei Xie, Alex Frano, Sohrab Ismail-Beigi, M. Brian Maple, and Yu He

Yu He
E-mail: yu.he@yale.edu
M. Brian Maple
E-mail: mbmaple@ucsd.edu,
Sohrab Ismail-Beigi
E-mail: sohrab.ismail-beigi@yale.edu

This PDF file includes:

Supporting text
Figs. S1 to S14
Tables S1 to S3
SI References

Supporting Information Text

Text S1. Tight Binding Model Fitting from ARPES

The electronic spectra of multilayer cuprates have been extensively studied using angle-resolved photoemission spectroscopy (ARPES), where the effective interlayer couplings (EICs) between different CuO_2 planes give rise to band splitting (1). Numerous studies have sought to quantify the strength of these EICs by fitting the observed band splitting into simplified tight-binding Hamiltonians, enabling the reproduction of experimental band dispersions based on these fits (2–5). For simplicity, the most prominent fitting for cuprate superconductors is a Cu-only framework that employs local orbitals with $d_{x^2-y^2}$ (d_{x^2}) symmetry. In prior literature, the EICs are usually described by an empirical formula

$$\Delta_{EIC} = t_0 + \frac{t_1}{4}\Big(\cos(k_x a) - \cos(k_y b)\Big)^2, \qquad [1]$$

where t_0 and t_1 are two fitting parameters. However, this formulation fails to capture essential spectral features observed in some multilayer cuprate systems (5).

In the main text, we successfully fit the ARPES spectrum with a more generic formula:

$$E_\pm = \epsilon_\pm - 2t_\pm(\cos k_x + \cos k_y) - 4t'_\pm \cos k_x \cos k_y - 2t''_\pm(\cos 2k_x + \cos 2k_y),$$

where $\pm$ represents antibonding (+) and bonding (-) bands, abbreviated as AB and BB. The band splitting $\Delta_{EIC} \equiv E_+ - E_-$ quantifies the strength of the interlayer coupling between the CuO_2 planes. To extract a tight-binding model, the formula can be reformulated as:

$$\begin{aligned} E_\pm = {} & \epsilon - 2t(\cos k_x + \cos k_y) - 4t' \cos k_x \cos k_y - 2t''(\cos 2k_x + \cos 2k_y) \\ & \pm \Big[t_{bi0} - 2t_{bi1}(\cos k_x + \cos k_y) - 4t_{bi2} \cos k_x \cos k_y - 2t_{bi3}(\cos 2k_x + \cos 2k_y)\Big], \end{aligned} \qquad [2]$$

where $\epsilon \equiv (\epsilon_+ + \epsilon_-)/2$ and $t_{bi0} \equiv (\epsilon_+ - \epsilon_-)/2$. Other parameters $(t, t', t''$, etc.) are defined analogously. The energies of AB and BB are distinguished by the $\pm$ sign in the formula. From Eq. (2), one can then read the following tight-binding model:

$$\begin{aligned} \hat{H} = {} & \sum_{il} \epsilon\, \hat{c}^\dagger_{il}\hat{c}_{il} - \sum_{\langle i,j\rangle l} t\, \hat{c}^\dagger_{il}\hat{c}_{jl} - \sum_{\langle\langle i,j\rangle\rangle l} t' \hat{c}^\dagger_{il}\hat{c}_{jl} - \sum_{\langle\langle\langle i,j\rangle\rangle\rangle l} t'' \hat{c}^\dagger_{il}\hat{c}_{jl} \\ & + \sum_{i\langle l,l'\rangle} t_{bi0}\hat{c}^\dagger_{il}\hat{c}_{il'} - \sum_{\langle i,j\rangle\langle l,l'\rangle} t_{bi1}\hat{c}^\dagger_{il}\hat{c}_{jl'} - \sum_{\langle\langle i,j\rangle\rangle\langle l,l'\rangle} t_{bi2}\hat{c}^\dagger_{il}\hat{c}_{jl'} - \sum_{\langle\langle\langle i,j\rangle\rangle\rangle\langle l,l'\rangle} t_{bi3}\hat{c}^\dagger_{il}\hat{c}_{jl'}. \end{aligned} \qquad [3]$$

Here i, j denote in-plane site indices, and l, l' are layer indices. NN, NNN, and 3NN pairs are represented by $\langle\cdot,\cdot\rangle$, $\langle\langle\cdot,\cdot\rangle\rangle$, and $\langle\langle\langle\cdot,\cdot\rangle\rangle\rangle$, respectively. Figure S1 illustrates this generic tight-binding model on a YBCO bilayer of CuO_2 planes. Black arrows indicate in-plane hoppings; blue arrows indicate interlayer hoppings.

Notably, the EICs in this model involve contributions from t_{bi1}, whose k-dependence is $\cos k_x + \cos k_y$, missing in the empirical formula Eq. (1). Recent studies have shown the importance of including this term in the fitting (6, 7). Microscopically, the effective t_{bi1} hopping is mediated by interlayer hopping between O p orbitals, which is large compared to t_{bi2} and t_{bi3} and cannot be ignored (7). Consistently, the magnitude of t_{bi1} fitted from experiment is larger than longer-ranged interlayer hoppings in the main text.

Text S2. DFT Calculation Details

Convergence of the Calculations. All crystal and electronic structure calculations in this study were performed using VASP (8, 9). Convergence is governed primarily by the plane-wave energy cutoff (ENCUT) and the k-point mesh density. We targeted convergence of energy differences within 1 meV per dopant atom. All calculations used EDIFF=10^{-6} eV and EDIFFG=-10^{-3} eV/Å. Gaussian smearing of 0.05 eV was applied for SCF.

Figure S2(a,b) show the lowest-energy and a metastable structure for distinct Ba-site Pr configurations. Figure S2(c–f) show convergence of their energy difference versus ENCUT and versus k-mesh density along three reciprocal directions. Based on these, we use ENCUT=500 eV and a $4 \times 6 \times 4$ mesh throughout, sufficient to converge energy differences to within 1 meV per Pr.

Magnetism and Effect of DFT+U Corrections. Like many hole-doped cuprates, $YBa_2Cu_3O_{7-\delta}$ exhibits competing magnetic orders on the CuO_2 planes (10); similar behavior occurs in $Bi_2Sr_2CaCu_2O_{8+x}$ (11). Using PBE+U with $U = 4$ eV, we identified many stripe-ordered states nearly degenerate with the G-AFM state. Figure S3 shows the spin densities for G-AFM and an example stripe state ("bond-centered" domain walls on O sites). The stripe state is only 1.2 meV per planar Cu above G-AFM. Planar Cu moments are $\sim 0.41\,\mu_B$ (G-AFM), $\sim 0.36\,\mu_B$ near the domain wall, and $\sim 0.46\,\mu_B$ away from it—consistent with prior DFT work on YBCO7 (10).

Because strong spin fluctuations are expected, a single ordered configuration is not an appropriate normal state. Prior work shows non-magnetic (NM) DFT bands agree well with ARPES Fermi surfaces (1, 12), providing a reasonable paramagnetic proxy (11). Thus, NM Cu was used for band-structure comparisons in the main text.

We employ DFT+U with $U = 4$ eV on Cu d orbitals to reduce self-interaction errors (13), following Refs. (7, 11, 14–16). The NM band structure in YBCO7 is only weakly affected by moderate changes of U (7), as illustrated in Fig. S4.

Stable or Metastable Crystals and Their Energies. Metastable structures were frequently encountered during relaxation. We focus on $Pr_{0.33}Y_{0.67}Ba_2Cu_3O_7$ (Y-site) and $Pr_{0.33}YBa_{1.67}Cu_3O_7$ (Ba-site). In a $3 \times 2 \times 1$ supercell, two Pr atoms replace Y or Ba. Figure S5 lists all relative positions investigated; all structures are fully relaxed. Planar Cu is set to NM; the two Pr spins are AFM-aligned. FM alignment is typically $\sim$1 meV/Pr higher, consistent with $T_N \approx 17$ K (17).

***f*-orbitals.** Following Liechtenstein–Mazin (18) for $PrBa_2Cu_3O_7$, we set $U_{\mathrm{Pr}} = 6$ eV and $U_{\mathrm{Cu}} = 0$ eV in the primitive cell (Fig. S6a). Figures S6(b,c) show the ground state with occupied $4f_{y(3x^2-y^2)}$ and $4f_{z^3}$ in spin-majority; other f orbitals are empty and ≥ 1 eV from E_F. Using occupation-matrix control (19) we explored 21 initial states; 18 distinct (meta)stable states were obtained (Table S1). The Fehrenbacher–Rice / Liechtenstein–Mazin configuration (18, 20) is reproduced but is metastable, 328 meV/Pr above the ground state (Figs. S6d,e).

Wannierization. Maximally localized Wannier functions (21) for Cu-d, O-p, and Pr-f were constructed using WANNIER90 (22). Figure S7 compares the unfolded VASP bands with the tight-binding bands from Wannierization; excellent agreement confirms high-quality projections.

Table S1. Different stable/metastable states and their total energy relative to the ground state, ΔE in meV per Pr atom. Each state is identified by the two occupied spin-majority $4f$ orbitals.

f-orbitals	$f_{y(3x^2-y^2)}, f_{z^3}$	$f_{z(x^2-y^2)}, f_{z^3}$	f_{xyz}, f_{z^3}	$f_{xyz}, f_{z(x^2-y^2)}$	$f_{y(3x^2-y^2)}, f_{xyz}$	$f_{y(3x^2-y^2)}, f_{z(x^2-y^2)}$
ΔE	0	328	196	482	189	523
f-orbitals	f_{yz^2}, f_{xyz}	$f_{z(x^2-y^2)}, f_{yz^2}$	$f_{xz^2}, f_{z(x^2-y^2)}$	$f_{xyz}, f_{z(x^2-y^2)}$	$f_{x(x^2-3y^2)}, f_{z^3}$	$f_{x(x^2-3y^2)}, f_{xyz}$
ΔE	491	158	330	489	1	192
f-orbitals	$f_{x(x^2-3y^2)}, f_{z(x^2-y^2)}$	$f_{xz^2}, f_{y(3x^2-y^2)}$	$f_{yz^2}, f_{y(3x^2-y^2)}$	$f_{yz^2}, f_{x(x^2-3y^2)}$	f_{yz^2}, f_{xz^2}	$f_{x(x^2-3y^2)}, f_{xz^2}$
ΔE	525	183	192	36	553	190

Table S2. Sample properties: doping level, T_c, and primary site occupancy.

Pr content (%)	0	5	15	12	28
onset T_c (K)	91	91	84	63	53
hole doping	0.31	0.34	0.22	0.18	0.11
site occupancy (SCXRD)	–	Y site	Y site	Ba site	Ba site
site occupancy (EDX)	–	Y site	Y site	Ba site	Y site (10%) and Ba site (18%)

Table S3. Tight-binding model fitting results (energies in meV).

sample	$\epsilon_\pm$	$t_\pm$	$t'_\pm$	$t''_\pm$
Pr 0% AB	435±2	218±1	-86 ± 1	24.4±0.2
Pr 0% BB	248±1	174±1	-83.2 ± 0.4	28.4±0.3
Type 1 5% AB	588±8	325±4	-133 ± 2	17.0±0.4
Type 1 5% BB	217±4	185±2	-88 ± 2	12.4±0.6
Type 1 15% AB	244±5	153±3	-51 ± 1	12.7±0.4
Type 1 15% BB	70±2	98±2	-39 ± 1	15±1
Type 2 12% AB	236±5	143±4	-54 ± 2	6±1
Type 2 12% BB	121±2	96±1	-30 ± 1	32±1

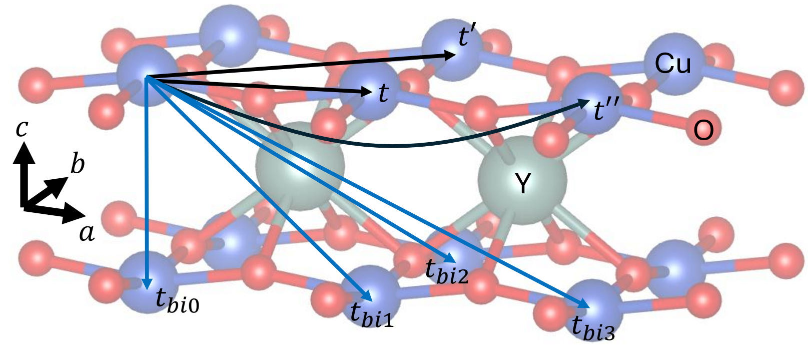

Fig. S1. Illustration of effective in-plane and interlayer hoppings in the tight-binding model of a YBCO CuO_2 bilayer.

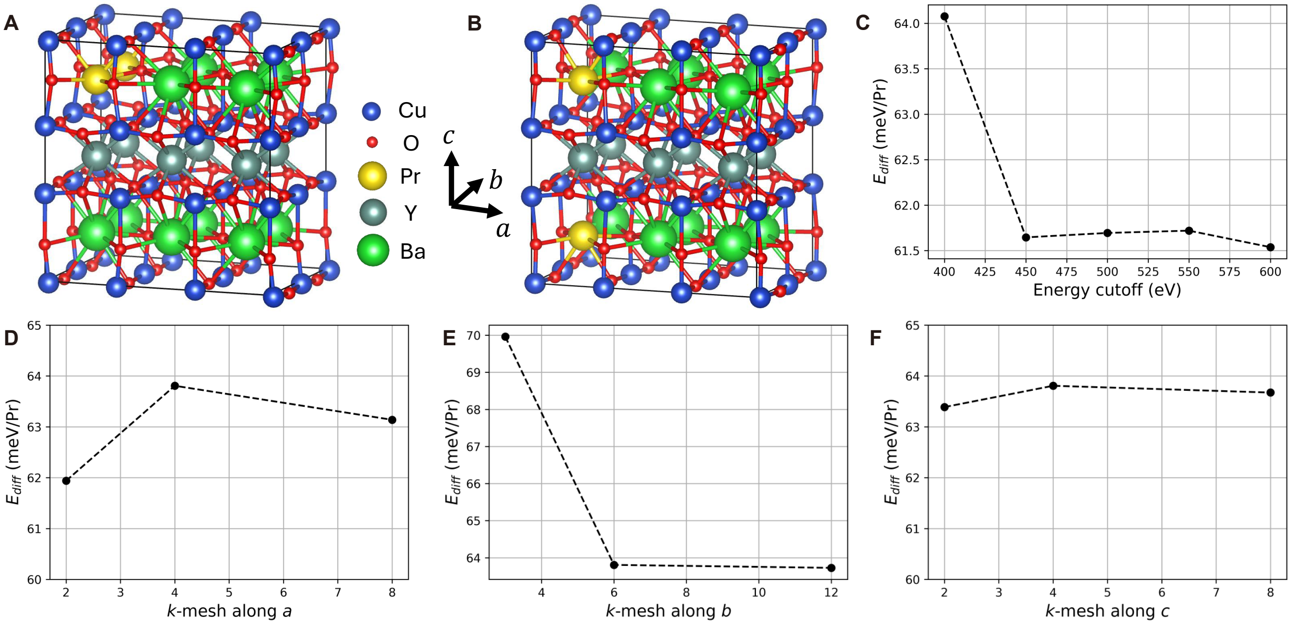

Fig. S2. Convergence of total-energy differences between dopant configurations vs. plane-wave cutoff and k-mesh density. (A) Ba-site Pr dopants aligned along b. (B) Ba-site Pr dopants aligned along c. (C) ΔE per Pr between (A) and (B) vs. cutoff E_c. (D–F) ΔE vs. k-mesh density along a–c. Default mesh is $4 \times 6 \times 4$ unless noted.

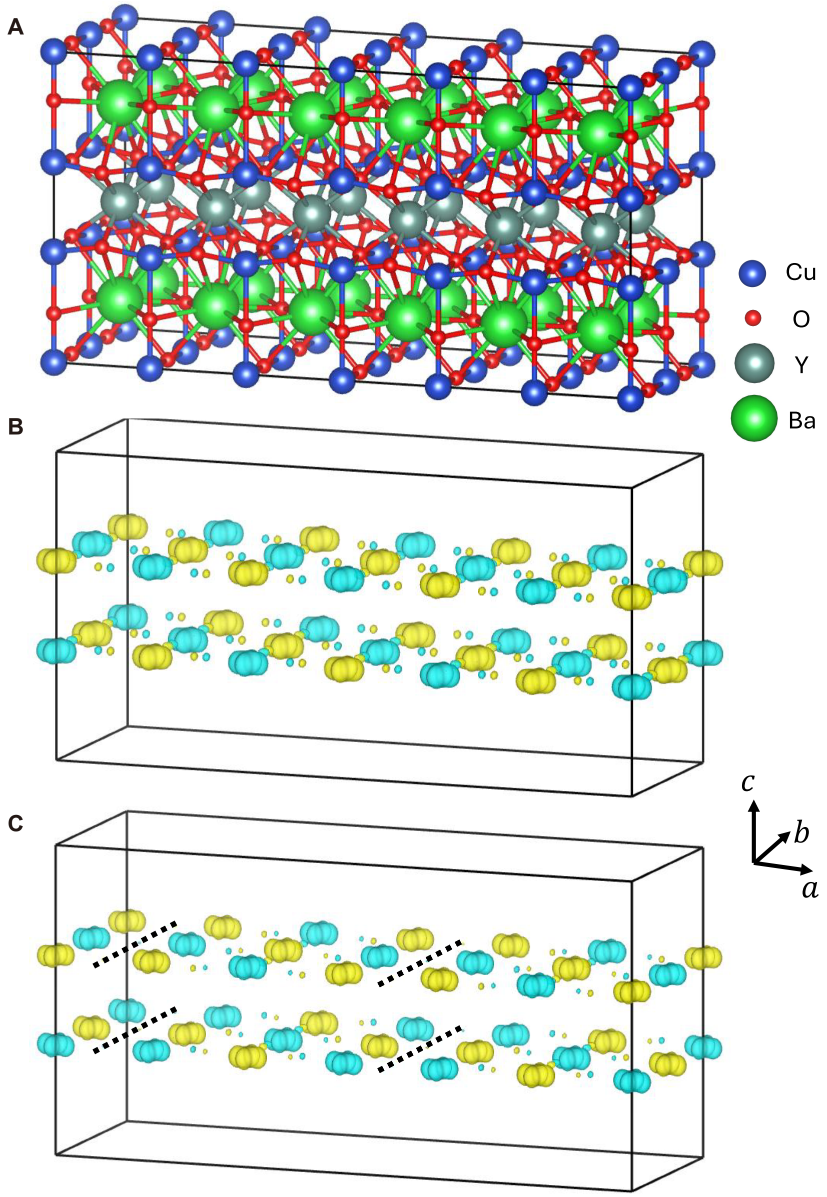

Fig. S3. (A) $6 \times 2 \times 1$ supercell. (B) Spin density isosurface of the G-AFM state. (C) Spin density isosurface of a stripe-ordered state; dashed lines mark domain walls.

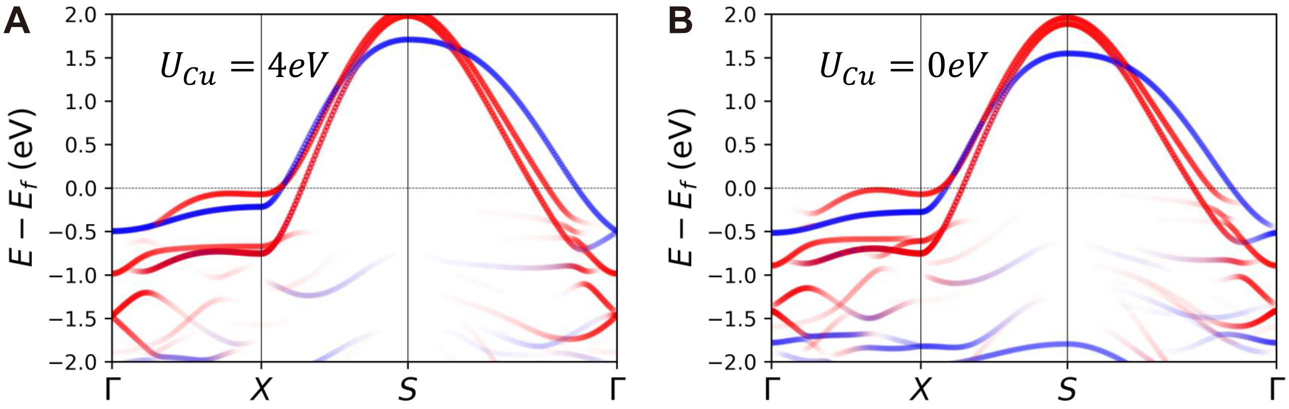

Fig. S4. (A) Projected bands of YBCO7 with DFT+U ($U = 4$ eV). (B) Projected bands without U. Red/blue indicate planar Cu $d_{x^2-y^2}$ / chain Cu d_{z^2}.

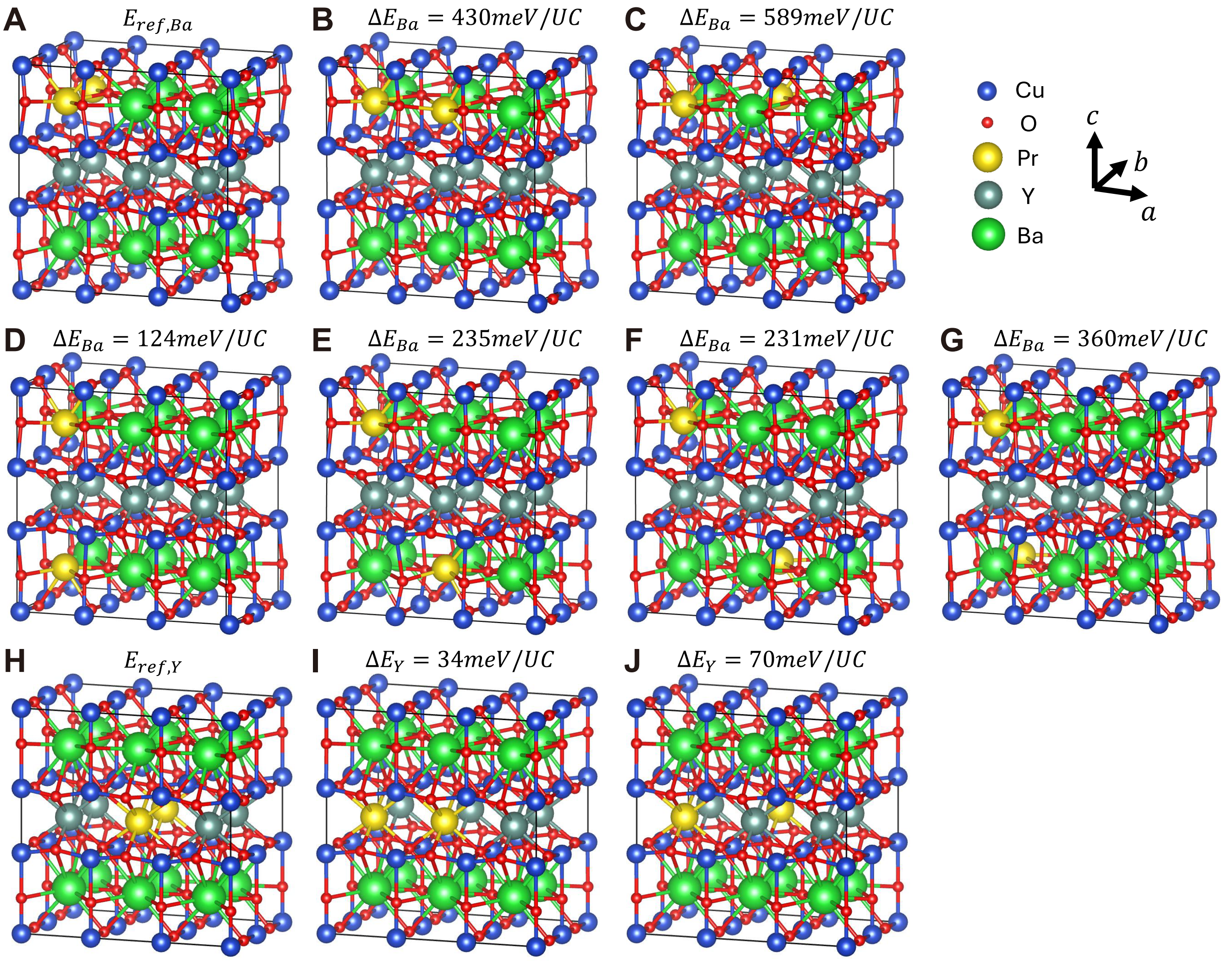

Fig. S5. Relaxed Pr dopant configurations in a $3 \times 2 \times 1$ supercell. (A) Ba-site Pr aligned along b; lattice $(a, b, c) = (11.45, 7.80, 11.73)$ Å (lowest energy, reference). (B–G) Other Ba-site metastable structures. (H) Y-site Pr aligned along b; $(11.54, 7.85, 11.85)$ Å (lowest Y-site energy). (I–J) Metastable Y-site structures.

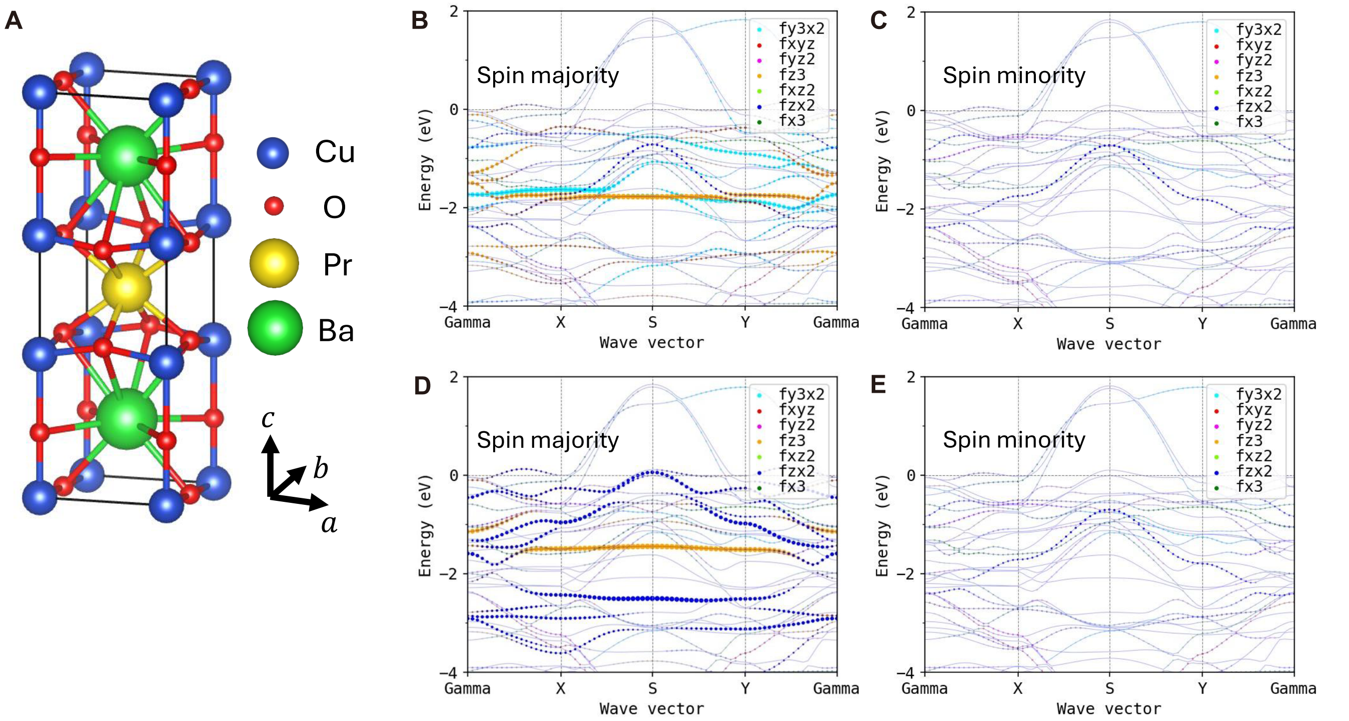

Fig. S6. (A) $PrBa_2Cu_3O_7$ primitive cell. (B–C) Ground-state spin-majority/minority: occupied $4f_{y(3x^2-y^2)}$ and $4f_{z^3}$. (D–E) Metastable FR/LM-like state with $4f_{z(x^2-y^2)}$ and $4f_{z^3}$; 328 meV/Pr above ground state.

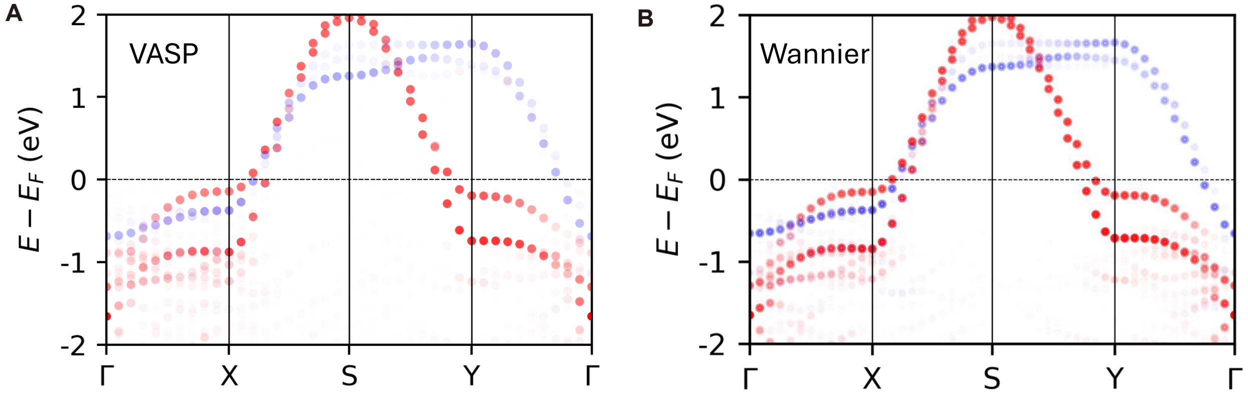

Fig. S7. Unfolded band structure of Ba-site Pr-doped YBCO7. (A) VASP. (B) Wannierized tight-binding. Opacity indicates orbital weight: red (planar Cu $d_{x^2-y^2}$), blue (chain Cu d_{z^2}), green (Pr f).

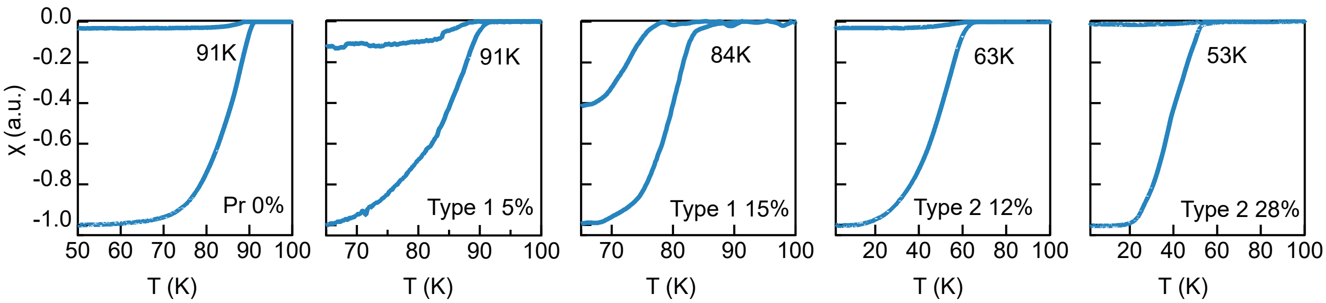

Fig. S8. Superconducting transition: magnetic moment under $H = 50$ Oe (out-of-plane).

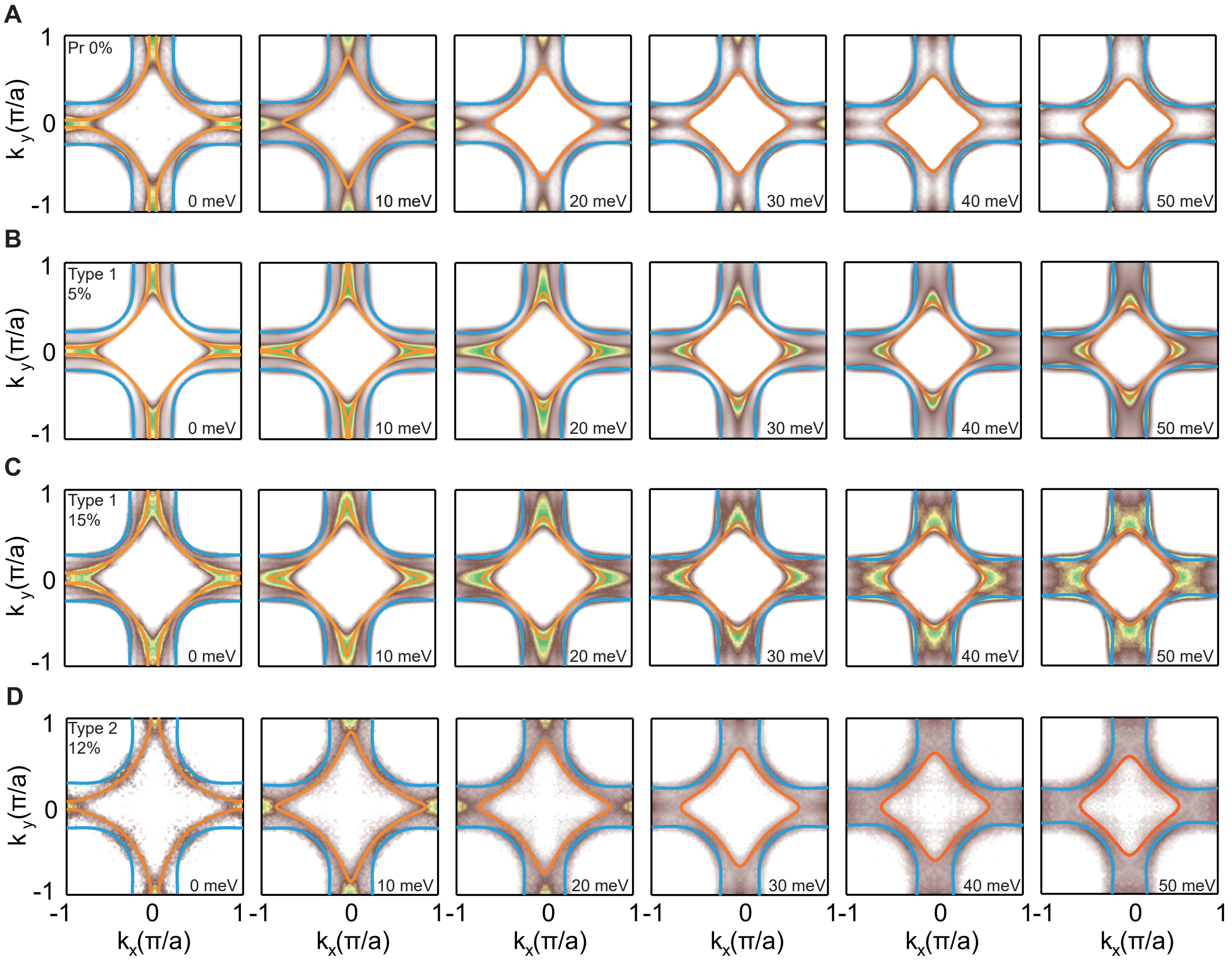

Fig. S9. Tight-binding fittings. Constant-energy maps for (A) Pr 0%, (B) Type-1 5%, (C) Type-1 15%, (D) Type-2 12% at 0–50 meV. Orange: BB k_F; cyan: AB k_F.

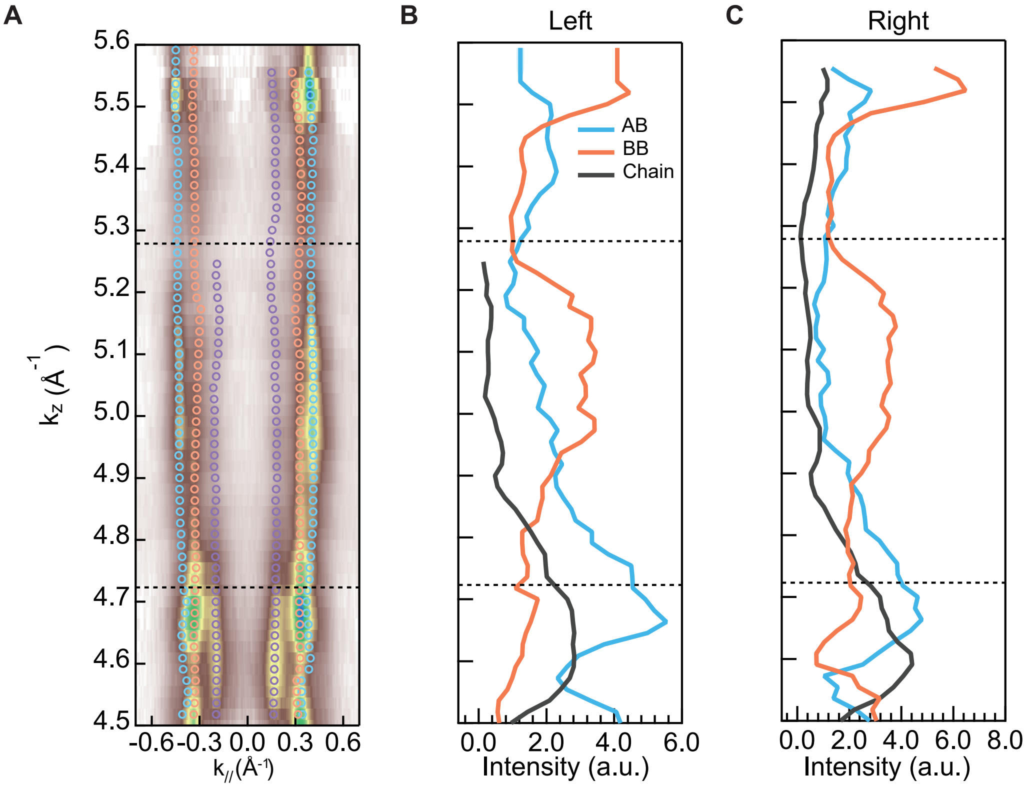

Fig. S10. k_z dispersion along the nodal direction (30–80 eV photons). (A) E_F map vs. $k_\parallel$ and k_z; MDC peak positions for AB (cyan), BB (orange), chain (purple). (B–C) Band-specific k_z intensity for left/right branches.

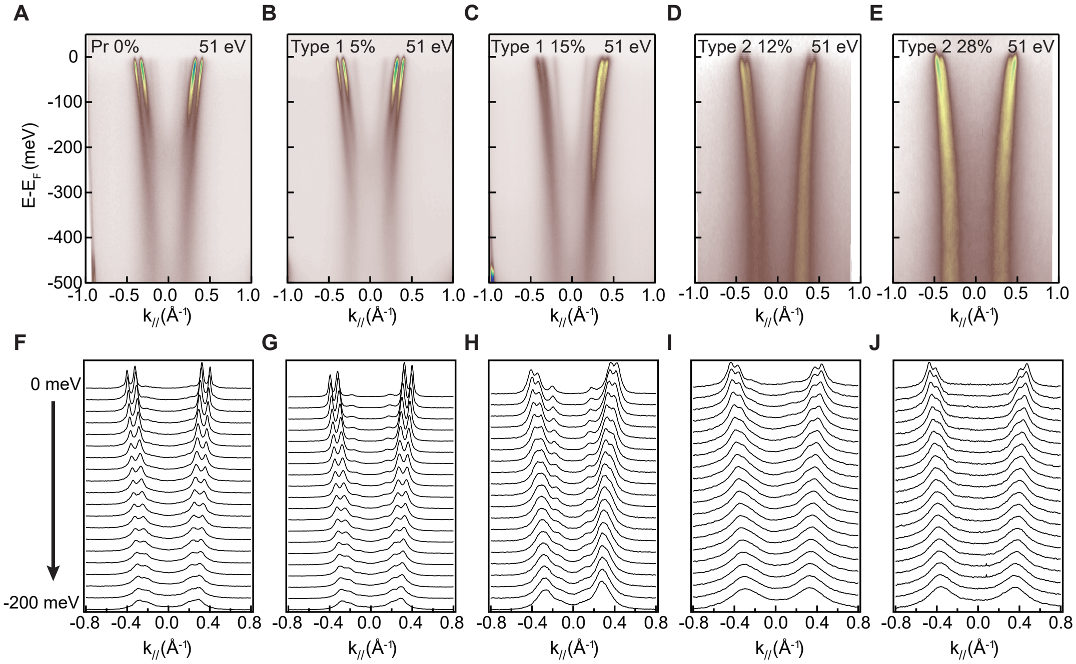

Fig. S11. (A–E) Nodal dispersions vs. photon energy highlighting plane bands for different Pr dopings. (F–J) Stacked MDCs from E_F to 200 meV.

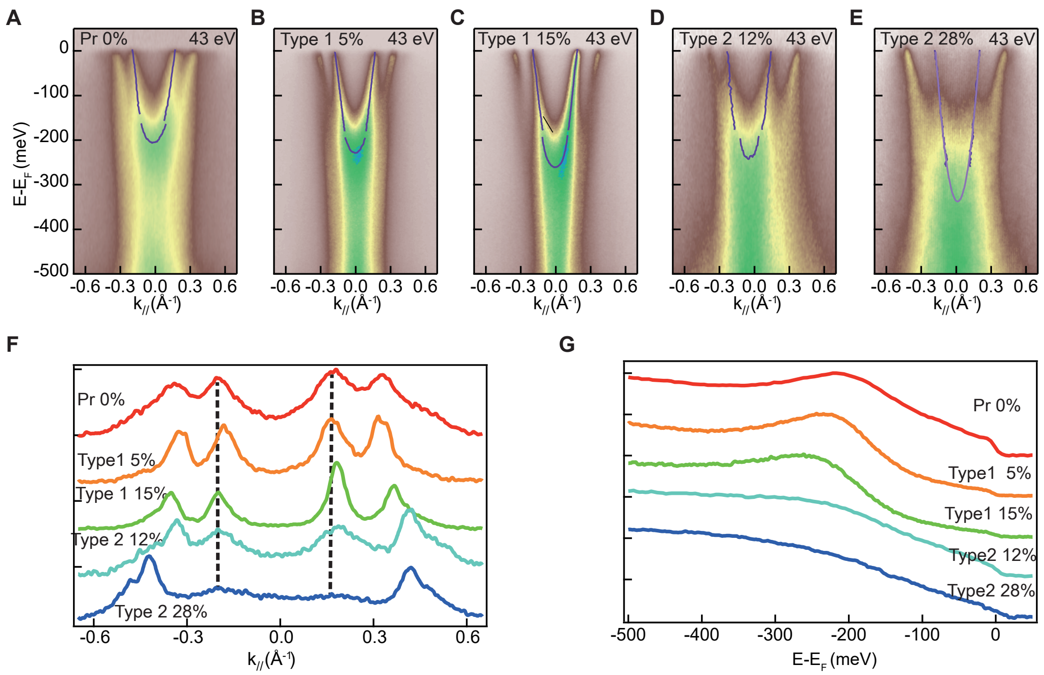

Fig. S12. (A–E) Nodal dispersions highlighting plane bands; fitted chain-band dispersion overlaid. (F) E_F MDCs. (G) EDCs at Γ.

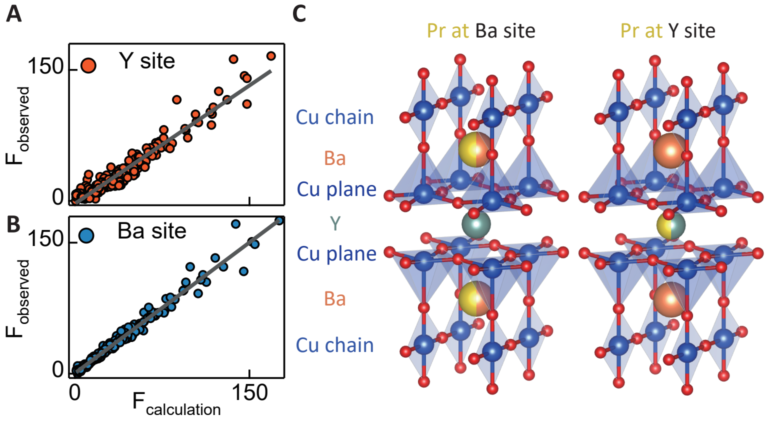

Fig. S13. Single-crystal XRD refinement of site occupancy. (A,B) Observed vs. refined structure factors for (A) Y-site and (B) Ba-site Pr substitution. (C,D) Structure models for (C) Y-site and (D) Ba-site substitution.

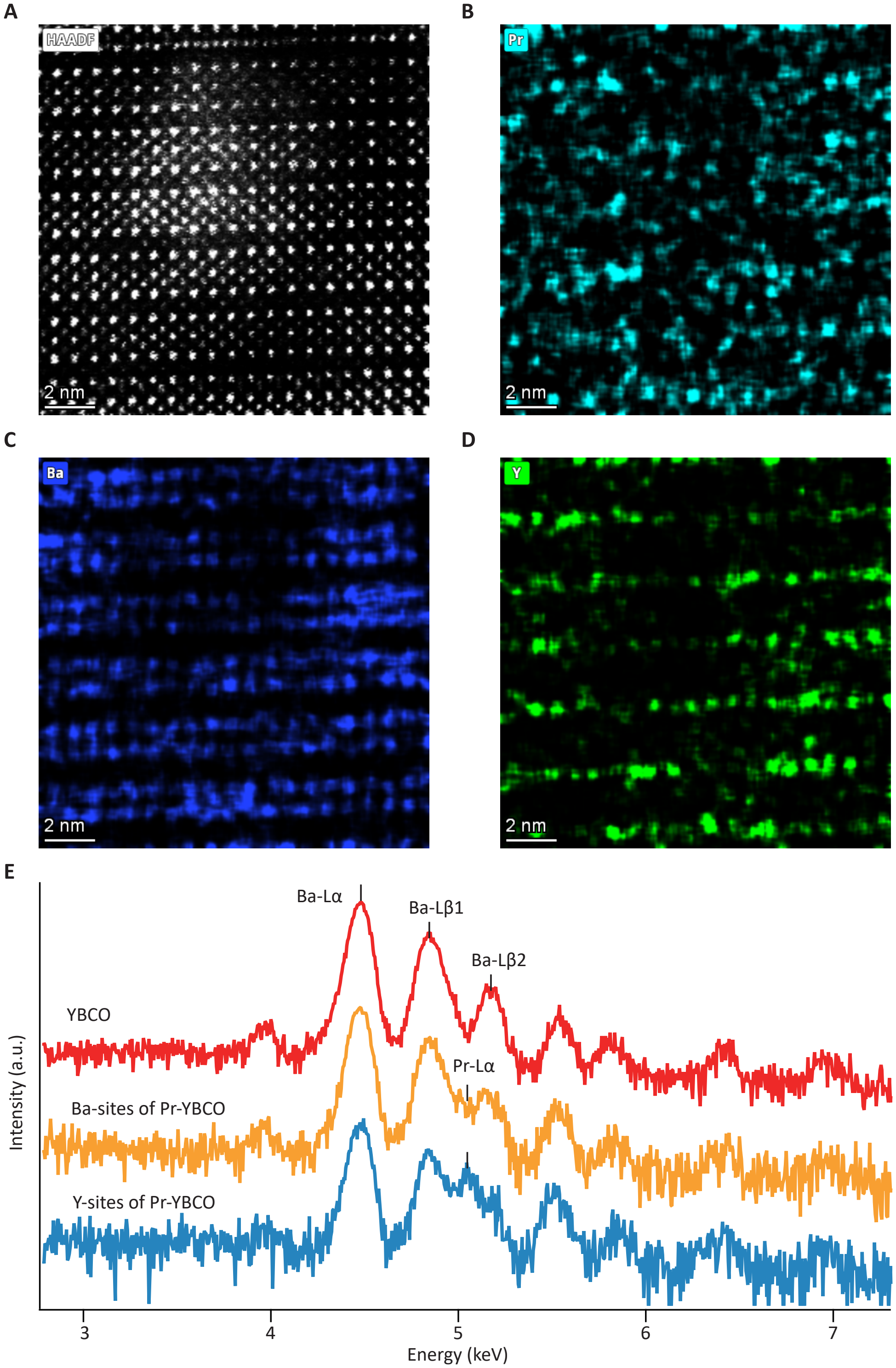

Fig. S14. HAADF-STEM and EDS for the Pr 12% substituted YBCO: (A) HAADF-STEM image; (B) Pr; (C) Ba; (D) Y EDS maps. (E) EDS spectra for pristine YBCO (red) and Pr-YBCO (orange/blue). Pr-Lα intensity appears at both sites, indicating Ba-site occupancy in addition to Y-site.

References

1. A Damascelli, Z Hussain, ZX Shen, Angle-resolved photoemission studies of the cuprate superconductors. *Rev. Mod. Phys.* **75**, 473–541 (2003).
2. S Chakravarty, A Sudbø, PW Anderson, S Strong, Interlayer tunneling and gap anisotropy in high-temperature superconductors. *Science* **261**, 337–340 (1993).
3. S Ideta, et al., Enhanced superconducting gaps in the trilayer high-temperature $Bi_2Sr_2Ca_2Cu_3O_{10+\delta}$ cuprate superconductor. *Phys. review letters* **104**, 227001 (2010).
4. S Ideta, et al., Hybridization of Bogoliubov quasiparticles between adjacent CuO_2 layers in the triple-Layer cuprate $Bi_2Sr_2Ca_2Cu_3O_{10+\delta}$ studied by Angle-Resolved Photoemission Spectroscopy. *Phys. Rev. Lett.* **127**, 217004 (2021).
5. X Luo, et al., Electronic origin of high superconducting critical temperature in trilayer cuprates. *Nat. Phys.* **19**, 1841–1847 (2023).
6. R Photopoulos, R Frésard, A 3D tight-binding model for La-based cuprate superconductors. *Annalen der Physik* **531**, 1900177 (2019).
7. Z Jin, S Ismail-Beigi, Interlayer couplings in cuprates: structural origins, analytical forms, and structural estimators (2024).
8. G Kresse, J Furthmüller, Efficiency of ab-initio total energy calculations for metals and semiconductors using a plane-wave basis set. *Comput. materials science* **6**, 15–50 (1996).
9. G Kresse, J Furthmüller, Efficient iterative schemes for ab initio total-energy calculations using a plane-wave basis set. *Phys. Rev. B* **54**, 11169–11186 (1996).
10. Y Zhang, et al., Competing stripe and magnetic phases in the cuprates from first principles. *Proc. Natl. Acad. Sci.* **117**, 68–72 (2020).
11. Z Jin, S Ismail-Beigi, First-principles prediction of structural distortions in the cuprates and their impact on the electronic structure. *Phys. Rev. X* **14**, 041053 (2024).
12. JA Sobota, Y He, ZX Shen, Angle-resolved photoemission studies of quantum materials. *Rev. Mod. Phys.* **93**, 025006 (2021).
13. JP Perdew, A Zunger, Self-interaction correction to density-functional approximations for many-electron systems. *Phys. Rev. B* **23**, 5048–5079 (1981).
14. C Yelpo, R Faccio, D Ariosa, S Favre, Electronic and vibrational properties of the high T_c superconductor $Bi_2Sr_2CaCu_2O_8$: an ab initio study. *J. Physics: Condens. Matter* **33**, 185705 (2021).
15. L Wang, T Maxisch, G Ceder, Oxidation energies of transition metal oxides within the GGA + U framework. *Phys. Rev. B* **73**, 195107 (2006).
16. L Deng, et al., Higher superconducting transition temperature by breaking the universal pressure relation. *Proc. Natl. Acad. Sci.* **116**, 2004–2008 (2019).
17. A Kebede, et al., Magnetic ordering and superconductivity in $Y_{1-x}Pr_xBa_2Cu_3O_{7-y}$. *Phys. Rev. B* **40**, 4453–4462 (1989).
18. AI Liechtenstein, II Mazin, Quantitative model for the superconductivity suppression in $R_{1-x}Pr_xBa_2Cu_3O_7$ with Different Rare Earths. *Phys. Rev. Lett.* **74**, 1000–1003 (1995).
19. JP Allen, GW Watson, Occupation matrix control of d-and f-electron localisations using DFT+ U. *Phys. Chem. Chem. Phys.* **16**, 21016–21031 (2014).
20. R Fehrenbacher, TM Rice, Unusual electronic structure of $PrBa_2Cu_3O_7$. *Phys. Rev. Lett.* **70**, 3471–3474 (1993).
21. N Marzari, D Vanderbilt, Maximally localized generalized Wannier functions for composite energy bands. *Phys. Rev. B* **56**, 12847–12865 (1997).
22. G Pizzi, et al., Wannier90 as a community code: new features and applications. *J. Physics: Condens. Matter* **32**, 165902 (2020).
